# Influence of gadolinium doping on structural, optical, and electronic properties of polymeric graphitic carbon nitride†

**DOI:** 10.1039/d4ra03437f

**Published:** 2024-07-24

**Authors:** Ganesh Kesavan, Dan C. Sorescu, Raihan Ahamed, Krishnan Damodaran, Scott E. Crawford, Faezeh Askari, Alexander Star

**Affiliations:** a Department of Chemistry, University of Pittsburgh Pittsburgh Pennsylvania 15260 USA astar@pitt.edu; b United States Department of Energy, National Energy Technology Laboratory Pittsburgh Pennsylvania 15236 USA; c Department of Chemical & Petroleum Engineering, University of Pittsburgh Pittsburgh Pennsylvania 15261 USA; d Department of Bioengineering, University of Pittsburgh Pittsburgh Pennsylvania 15261 USA

## Abstract

Polymeric graphitic carbon nitride (gCN) materials have received great attention in the fields of photo and electrocatalysis due to their distinct properties in metal-free systems with high physicochemical stability. Nevertheless, the activity of undoped gCN is limited due to its relatively low specific surface area, low conductivity, and poor dispersibility. Doping Gd atoms in a gCN matrix is an efficient strategy to fine-tune its catalytic activity and its electronic structure. Herein, the influence of various wt% of gadolinium (Gd) doped in melon-type carbon nitride was systematically investigated. Gadolinium-doped graphitic carbon nitride (GdgCN) was synthesized by adding gadolinium nitrate to dicyandiamide during polymerization. The X-ray diffraction (XRD) and transmission electron microscopy (TEM) results revealed that the crystallinity and the morphological properties are influenced by the % of Gd doping. Furthermore, X-ray photoelectron spectroscopy (XPS) studies revealed that the gadolinium ions bonded with nitrogen atoms. Complementary density functional theory (DFT) calculations illustrate possible bonding configurations of Gd ions both in bulk material and on ultrathin melon layers and provide evidence for the corresponding bandgap modifications induced by gadolinium doping.

## Abstract 2

Synthesis of gadolinium-doped graphitic carbon nitride *via* thermal polymerization and study of its electronic properties.

## Graphical abstract

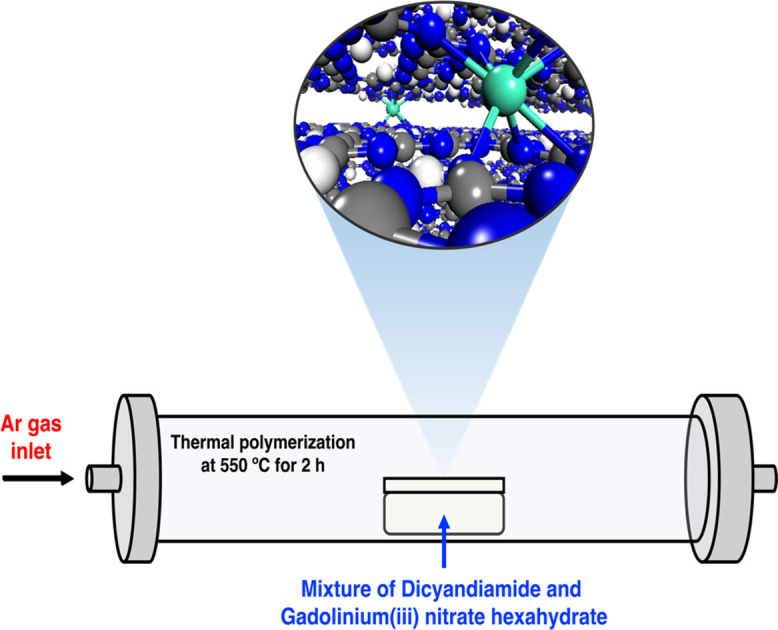

## 1. Introduction

Two-dimensional (2D) materials such as transition metal dichalcogenides (TMDs), layered double hydroxides (LDHs), metal carbides and nitrides (collectively known as MXenes), boron nitrides, hexagonal boron nitride (h-BN), polymeric carbon nitride, black phosphorus (or phosphorene), arsenene, antimonene and bismuthine have received great attention in field-effect transistors, thermoelectrics, topological insulators, nanoelectronics, optoelectronics, and spin- and valley-tronics.^1,2^ These materials are sheet-like structures of a single or few atoms thick, ranging from a few nanometers to several hundreds of nanometers with unique bonding interactions.^3^ Notably, polymeric graphitic carbon nitride (gCN) is a layered material composed of earth-abundant carbon (C) and nitrogen (N) elements and is characterized by an optical bandgap (*E*_opt_) of ∼2.7 eV.^4^ The fully condensed form of carbon nitride with the generic formula of g-C_3_N_4_ is characterized as a stable phase having a highly corrugated layered structure. It is highly chemically stable in both acidic and alkaline environments and thermally stable up to 600 °C in ambient atmosphere due to the tri-*s*-triazine ring structure.^5,6^ Due to its low toxicity, ease of preparation, biocompatibility, and chemical inertness, gCN has broad interdisciplinary applications including energy conversion and storage, H_2_ production, adsorption, sensing, membranes for gas separation, photocatalysis, biomedicine, and ammonia reduction.^7,8^ Additionally, the synthesis of gCN is relatively straightforward and involves condensation pathways that can be initiated from a variety of nitrogen-rich compounds like dicyandiamide, urea, melamine, and thiourea.^9^ However, the efficiency of such condensation processes is limited as it often leads to uncondensed or melon-type carbon nitride (C_6_N_9_H_3_, gCN).^10,11^ It is challenging to synthesize fully condensed graphitic carbon nitride with a C/N ratio of 0.75, as significant amounts of hydrogen atoms or NH_2_ functional groups are present in the gCN structure,^12^ features that in turn can have a negative impact upon the specific surface area, conductivity, and material dispersibility. To overcome such limitations, several methods have been developed including doping with metallic and non-metallic elements or interfacing with other semiconductors and carbonaceous materials.^13^ Changes in porosity and exfoliation are other possible techniques to enrich the efficacy of the gCN catalysts and to modulate the corresponding electronic properties.^13^ Among several known techniques, the most promising approach for controlling the physicochemical properties is through metal doping.^14^ This strategy can effectively improve the electron transport mobility across the structure and significantly fine-tune the gCN bandgap. In addition, metallic dopants can also act as catalysts or co-catalysts during reactions, therefore increasing the overall performance. Thus, resulting properties can be brought into play to enhance a wide range of applications such as N_2_ reduction reaction,^15,16^ photocatalytic applications,^17^ CO_2_ reduction,^18^ and photocatalytic H_2_ evolution.^19^

Lanthanide (Ln) materials are widely used in several scientific and industrial applications because of their well-defined physicochemical properties.^20^ Doping effects greatly depend upon the nature of the Ln ions with systems having 3+ oxidation states being more customizable as their fluorescence levels encompass UV-vis-NIR regions.^21,22^ Gadolinium is a particularly versatile dopant among the lanthanides: gadolinium-based nanoparticles have been employed in several medical and imaging applications.^23^ Such systems have been demonstrated to provide excellent contrast efficiency, as the gadolinium-based contrast agent (GBCA) is considered one of the safest contrasting agents.^24^ The influence of Ln elements doping in gC_3_N_4_ materials has been previously investigated. For instance, Liu *et al.* incorporated terbium ions into the gCN framework using microwave irradiation at 800 W for 3 min.^25^ The doped sample displayed multi-emission fluorescence at 490 and 545 nm upon 290 nm excitation. The results conveyed that the wavelengths of emission maxima and the fluorescence intensities can be adjusted based on the concentrations of doping ions.^25^ Mori *et al*. prepared europium-doped exfoliated g-C_3_N_4_ nanosheets (Eu/nanoC_3_N_4_) with ammonium chloride as a dynamic gas template.^26^ The results demonstrated that single-atom Eu^3+^ ions were successfully doped in an exfoliated g-C_3_N_4_ nanosheet framework. Similarly, Li *et al.* synthesized bayberry-like hollow Gd/g-C_3_N_4_ nanospheres *via* a hard template solvothermal process using SiO_2_ nanospheres. The prepared material with 2 wt% Gd achieved enhanced photocatalytic performance and a photodegradation efficiency of Rhodamine B (RhB) that is 2.12 times higher than pure g-C_3_N_4_.^27^

The adsorption capacity of gadolinium ions (Gd^3+^) on gC_3_N_4_ was also investigated by Kuila *et al.*^28^ Urea was used in this case for the synthesis of gC_3_N_4_, and the gC_3_N_4_ was then used as an adsorbent for gadolinium ions (Gd^3+^) from aqueous solution. The adsorption capacity of g-C_3_N_4_ was found to be influenced by several parameters including the initial Gd^3+^ concentration, solution pH, and the contact time.^28^

It is of interest to expand upon these previous studies and to explore the case when doping with lanthanide ions is performed for the case of a melon network. For this purpose, the present study addresses the influence of gadolinium doping in the melon phase of carbon nitride. The corresponding structural, optical, and electronic properties are evaluated *via* several experimental techniques including X-ray diffraction (XRD), X-ray photoelectron spectroscopy (XPS), optical absorption, and solid-state nuclear magnetic resonance (ssNMR) techniques. Although experimental data identify the main features of the impact produced by Gd doping upon gCN structure, the interpretation of such complex experimental results is challenging. As a result, complementary theoretical studies were carried out to investigate the properties of the dopants inside the melon matrix and to provide additional support in the interpretation of the structural and spectroscopic results obtained.

## 2. Experimental section

### 2.1. Materials and methods

All reagents in this study were of analytical grade and used without any purification process. De-ionized water (DIW, 18.2 MΩ cm^−1^) was used throughout the experiments. Dicyandiamide (C_2_H_4_N_4_, DCDA), and gadolinium(iii) nitrate hexahydrate Gd(NO_3_)_3_·6H_2_O, were purchased from Sigma Aldrich.

The preparation of melon-type carbon nitride was carried out using a solvent evaporation technique followed by direct thermal polymerization.^29^ About 1 g of DCDA was measured and dispersed in 10 mL of nanopure water at 80 °C. A varying weight ratio (*x* = 1, 3, 5, and 10 wt%) of Gd(NO_3_)_3_·6H_2_O was added to the DCDA solution and allowed to stir until the solvent completely evaporated. The resultant white powder was transferred to a 50 mL alumina crucible covered with a lid and polymerized at 550 °C for 2 h under an argon (Ar) atmosphere. For pristine gCN, the above procedure was repeated without adding Gd(NO_3_)_3_·6H_2_O precursor.

### 2.2. Characterization techniques

The prepared materials were characterized using the following techniques. Powder X-ray diffraction (XRD) patterns of the prepared samples were obtained using D8 DISCOVER, Bruker Cu-Kα irradiation (wavelength: 1.5406 Å) at 40 kV and 40 mA. A scan rate of 0.1° s^−1^ was applied to record the powder XRD patterns for 2*θ* in the range of 10–70°. Attenuated Total Reflectance-Fourier Transform Infrared (ATR-FTIR) spectra were recorded between 4000 and 400 cm^−1^ using an ALPHA II Compact FT-IR Spectrometer.

The surface morphology of the samples was analyzed using a transmission electron microscope (TEM, HT7800 series) at an accelerating voltage of 300 kV. Excitation and emission spectra were analyzed using a HORIBA Jobin-Yvon Fluorolog 3 equipped with FluorEssence software and a 450 W xenon arc lamp. A 365 nm excitation wavelength was used for all measurements with excitation and emission slits set to 2 nm. A 0.1 s integration time was used, and all spectra were corrected for gratings, lamp, and detector response. A 380 nm cut-off filter (Edmund Optics) was used during all measurements, and a water blank was subtracted from all spectra. Quartz cuvettes (Thorlabs) were used for all photoluminescence and absorption measurements. Absorption spectra were recorded using a Thermo Evolution 600 UV-vis spectrophotometer. All the samples were sonicated for 1 h and kept at rest for 30 minutes. The supernatants were collected for taking the absorption measurements. X-ray photoelectron spectroscopy (XPS) data were obtained on a Thermo Fisher ESCALAB 250 Xi XPS instrument with a monochromatized Al Kα line source (150 W) with ion beam etching for 10 s. The XPS deconvolution was carried out using XPS peak41 software by applying a Shirley-type background subtraction. All binding energies were referenced to the C 1s peak at 284.8 eV. Solid-state NMR experiments were performed on a Bruker Avance-I 600 MHz spectrometer operating at a magnetic field strength *B*_0_ of 14.1 T, with ^1^H/^13^C/^15^N Larmor frequencies of 600.6/151.0/60.9 MHz, respectively. All the experiments were performed using a Bruker 3.2 mm probe at ambient conditions. The proton longitudinal relaxation was measured using inversion recovery with ^13^C CP detection with the standard Bruker pulse sequence cpht1.av. ^13^C NMR chemical shifts were reported relative to TMS (0 ppm), referenced using a secondary reference of glycine (C=O at *d* = 176.5 ppm). ^15^N NMR chemical shifts were referenced using a secondary reference of glycine assigned to 33.4 ppm, relative to liquid NH_3_.

### 2.3. Computational methods

The structural and electronic properties of Gd doped in bulk melon or in ultrathin melon nanostructures containing 1–2 layers have been investigated using spin-polarized calculations within density functional theory as implemented in the Quickstep module of CP2K code.^30,31^ This package uses a mixed gaussian and plane-wave basis set in combination with Goedecker–Teter–Hutter pseudopotentials.^32^ A TZVP-MOLOPT triple-zeta basis set^33^ with one set of polarization functions was used for all atoms together with a plane-wave basis set with an energy cutoff of 1000 Ry. Large supercell models containing C_192_H_96_N_288_ composition were used for bulk calculations and optimizations were performed in these cases at the *Γ k*-point using Perdew–Burke–Ernzerhof (PBE)^34^ exchange correlation functional augmented with Grimme D2 method to include long-range dispersion interactions.^35,36^ For the optimized structures, evaluation of the electronic properties was performed using Heyd, Scuseria, Ernzerhof (HSE06)^37,38^ hybrid functional with 25% exact exchange which was shown to describe accurately the optical properties of carbon nitride structures.^39^ For hybrid functional calculations, reduction in computational cost has been obtained using the auxiliary density matrix method (ADMM) of Guidon *et al.*^40,41^ to approximate the exact exchange contribution. For this purpose, the auxiliary basis sets pFIT3 for non-metal atoms and FIT14 for Gd as developed by S. Ling and B. Slater and implemented in cp2k code have been used.^30,31^

## 3. Results and discussion

### 3.1. XRD and FTIR analysis

**Fig. 1:**
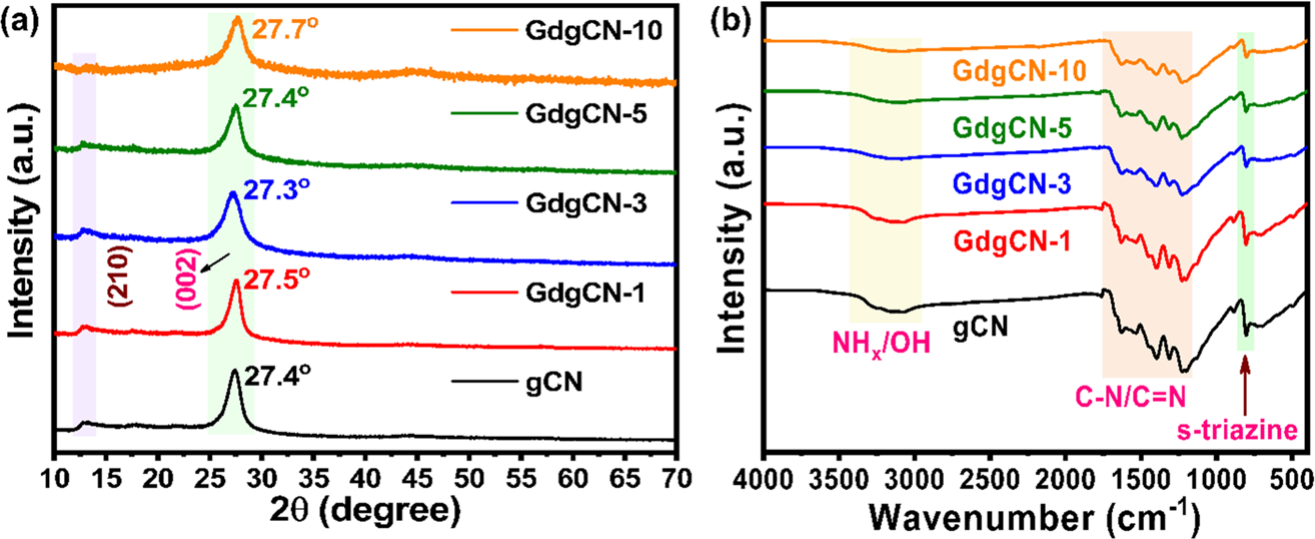
(a) XRD and (b) FTIR patterns of synthesized gCN and GdgCN-*x* (*x* = 1, 3, 5, and 10 wt%) samples.

The powder XRD patterns of the prepared materials obtained from the thermal polymerization of DCDA and Gd(NO_3_)_3_·6H_2_O are shown in Fig. 1a. All samples display similar XRD patterns, in which pristine or undoped gCN shows the strongest reflection peak at 2*Θ* ≈ 27.4° corresponding to the (002) diffraction plane that originates from the periodic stacking of melon nanosheets along the *z*-axis. A weak reflection peak at 2*Θ* ≈ 12.8° which corresponds to the (210) plane indicates the 1D polymeric chains of heptazine units along the *xy*-plane.^42^ The smaller peaks at 2*Θ* ≈ 17.8°, 21.4°, 44.5°, and 56.4° were assigned to in-plane diffractions from the (310), (320), (300), and (004) planes, respectively.^43,44^ The XRD peaks of the GdgCN_*x*_ samples with varying wt% of Gd were found to be similar to those of gCN. A significant shift in the (002) diffraction plane to lower and higher angles was observed with a decrease in intensity, and no other diffraction peaks related to metallic Gd are noted, suggesting that Gd ions were incorporated in-between gCN layers.^45^ A possible reason for this trend could be the incorporation of Gd atoms without lattice distortion. The substitution of Gd ions might not cause a significant distortion in the crystal structure. This minimal distortion might not translate into major peak shifts in the XRD pattern.^46^ Relative to pristine gCN XRD spectra, with the increase of the Gd content, the intensity of the peak at 12.8° decreases which indicates a reduction of the long-range order along the *xy* plane due to the rupture of intralayer H-bonding.^47^

The functional chemical groups of various prepared samples were examined using ATR-FTIR and the corresponding results are shown in Fig. 1b. The pristine gCN displays a strong peak at 806 cm^−1^ corresponding to the out-of-plane bending vibrations of the tri-*s*-triazine units. The sequence of the peaks from 1200 to 1700 cm^−1^ corresponds to the stretching vibration modes of C–N and C=N heterocycles. The region between 3000–3500 cm^−1^ is assigned to the stretching vibrations of the amine group (NH_*x*_) or OH groups.^48,49^ Overall, for the entire set of Gd concentrations no considerable decrease in peak intensity and no observable change in peak position with increasing Gd content is observed suggesting that no changes in gCN structure take place. It is worth noting that the vibrational peaks of Gd ions were not observed, which could be due to the strong overlap with the C–N vibrations. At higher temperatures, previous studies indicated the possibility that Gd(NO_3_)_3_·6H_2_O will lead to the formation of GdO metal oxide.^50^ However, in the current work, no such peaks related to GdO formation were observed.

### 3.2. Photoluminescence and UV-vis analysis

**Fig. 2:**
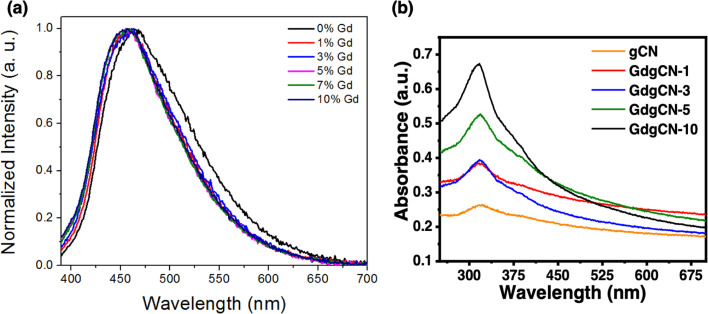
(a) Photoluminescence and (b) UV-vis absorption spectra of gCN and GdgCN-*x* samples.

To further investigate the effect of gadolinium doping upon optical properties, the photoluminescence (PL) spectra were analyzed, and the corresponding results are shown in Fig. 2a. All the samples were measured at room temperature after sonication in water under an excitation wavelength of 365 nm. The emission peak of pristine gCN was observed at 463 nm which is attributed to the band–band PL phenomenon. From Fig. S1,† the PL spectrum of gadolinium nitrate hexahydrate displays a high-intensity peak at 436 nm. Upon increasing Gd concentrations, the shape of the PL curves remained similar to that of gCN and the emission wavelengths underwent a blue shift to 456 nm for 10 wt% sample (Fig. S2a†). The obtained *λ*_max_ values are summarized in Table S1.† The PL spectrum of a previously reported melon sample prepared *via* a solvent evaporation pre-treatment technique was centered at 463 nm.^29^

The light absorption properties of the prepared gCN and GdgCN-*x* samples were analyzed using UV-vis spectroscopy. All the samples were sonicated for 1 h before taking the absorption spectra. The exfoliated melon nanosheets were then collected from the top of the suspension after keeping the samples at rest for 30 minutes. From Fig. 2b, it can be seen that all the samples show a maximum absorption within the 320–330 nm region, and a spectral tail extended in the visible range. Higher values of absorbance with increased content of Gd are observed due to the presence of higher concentrations of melon nanosheets in the suspensions, indicating that the incorporation of Gd atoms into the gCN structure helps to better exfoliate into melon nanosheets during the sonication process. In addition to the fundamental absorption peak, a shoulder absorption peak is observed for each sample which represents the defect states present within the bandgap of melon. The optical bandgap energy *E*_opt_ values for the gCN samples doped with different amounts of Gd were further calculated using a Tauc plot (Fig. S3†). For the gCN sample, the corresponding bandgap was determined to be 3.1 eV which is less than the electronic bandgap (*E*_g_) values previously reported for melon.^42,51^ The *E*_opt_ values for melon are generally less than the *E*_g_ values because a part of the *E*_g_ is consumed to generate separated charge carriers from a tightly bound electron–hole pair, known as the exciton binding energy.^32^ The *E*_opt_ value obtained from the absorbances due to the defect states of melon is 2.67 eV which matches the *E*_opt_ value calculated from the PL spectra.

After Gd doping, the samples display similar peaks with gCN and increasingly larger *E*_opt_ values up to 3.4 eV for 10 wt% sample. The increase in the bandgap could be due to the quantum confinement effect present in ultrathin melon nanosheets.^32^ The formation of such nanosheets could be enhanced in the case of intercalated Gd atoms due to the extra stresses imposed upon the melon framework by the doped atoms which in turn can help to exfoliate bulk melon to thin sheets during the sonication process. This restricts the motion of charge carriers due to quantum confinement, which increases their corresponding bandgaps.^52,53^

### 3.3. Morphological analysis

**Fig. 3:**
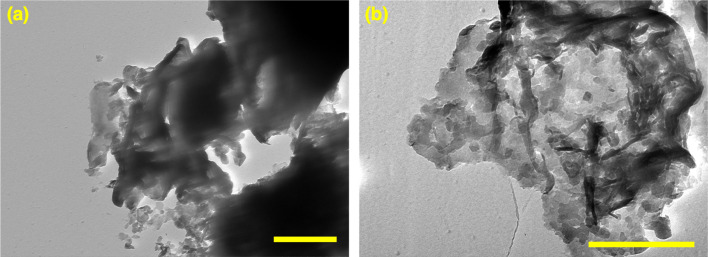
TEM images of exfoliated (a) gCN and (b) GdgCN-10 samples (scale bar = 500 nm).

The morphology of the exfoliated gCN and GdgCN-*x* samples was investigated by transmission electron microscopy (TEM). From Fig. 3a, it can be seen that the gCN sample exhibits irregular sheets that are several nanometers in length. The addition of Gd precursor significantly influenced the morphological evolution as thinner and smaller size sheets were observed as depicted in Fig. 3b. The presence of gadolinium precursor generates nitrogen oxide and water during the polymerization process,^50^ which in turn can act as a template to modify the final texture. It has been indicated in the previous section that gadolinium doping assists in the exfoliation of carbon nitride melon sheets upon sonication when preparing samples for analysis by optical spectroscopy (*i.e.*, UV-vis absorption and photoluminescence). The decrease in melon sheet thickness can be correlated to the observed increase in the measured direct bandgap of the gadolinium-doped samples. It is worth noting that TEM analysis did not reveal any evidence of particle formation on the surface of GdgCN-*x* samples, indicating gadolinium ion intercalation between melon layers (*vide infra*). For comparison, the TEM images of bulk gCN and GdgCN-*x* samples before exfoliation are displayed in (Fig. S4†).

### 3.4. XPS analysis

**Fig. 4:**
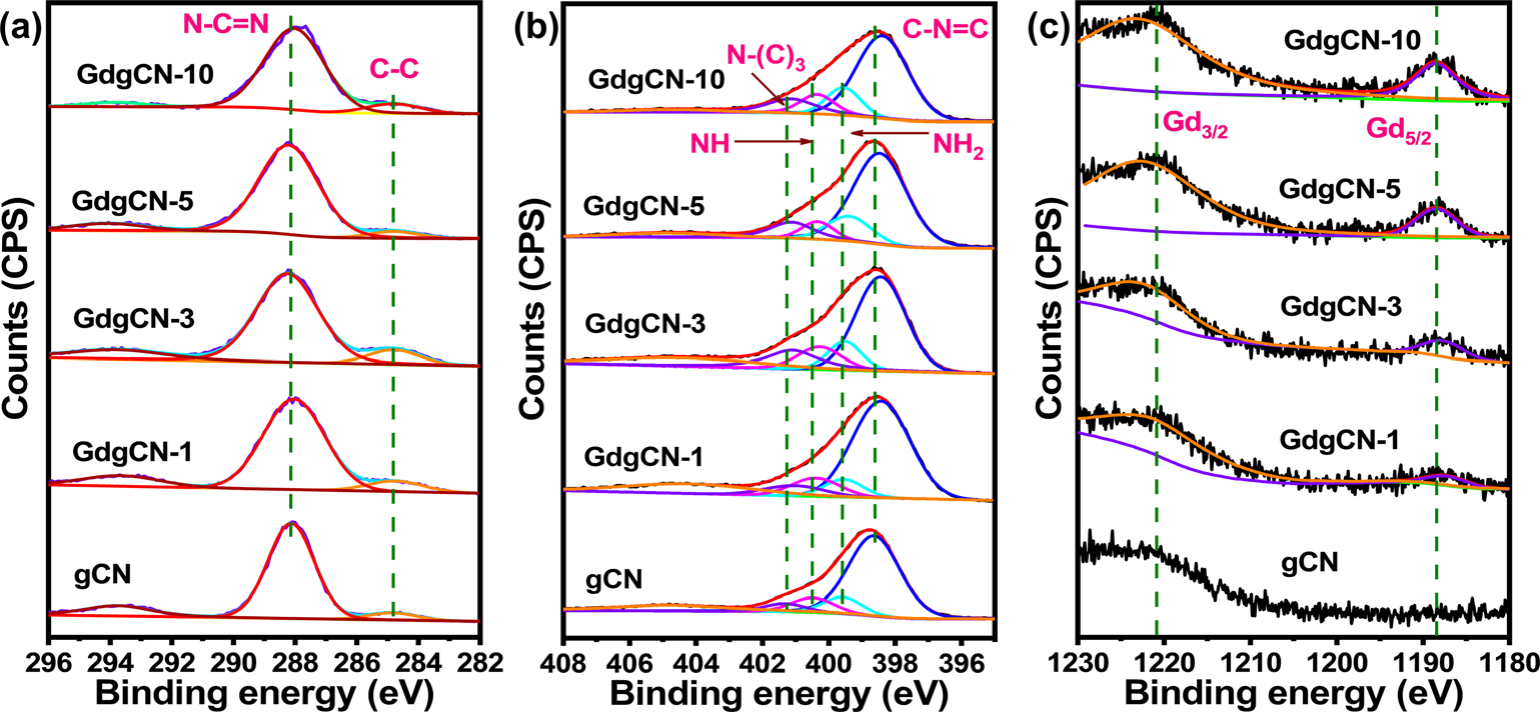
(a) C 1s spectra, (b) N 1s spectra, (c) Gd 3d spectra of gCN and GdgCN samples.

The electronic structure of gCN and various Gd-doped gCN samples were characterized using XPS analysis. The C 1s, N ls, O 1s, and Gd 3d levels were recorded for all the materials for comparative purposes. From the wide-scan XPS spectrum of the gCN sample, the presence of carbon (C), nitrogen (N), and oxygen (O) elements can be seen, whereas Gd-varied samples show Gd peaks between 1170–1240 eV (Fig. S5†). The small amount of oxygen observed in all samples corresponds to adsorbed water and CO_2_ from atmosphere.^54^ From Fig. 4a, the C 1s spectrum of gCN shows two main peaks at binding energies of 284.8 eV and 288.4 eV. The peak at 284.8 eV can be attributed to adventitious carbon (AdC) which is used as a calibration peak. The binding energy at 288.2 eV is assigned to sp^2^ bonded carbon (N–C=N). Remarkably, C 1s for Gd-doped samples show similar peaks with less intensity. The N 1s spectra of gCN in Fig. 4b were deconvoluted to four peaks at 398.54, 399.42, 400.23, and 401.23 eV corresponding to sp^2^-hybridized N involved in triazine rings (C–N=C), primary amines (NH_2_), pyrrole type (NH), and tertiary nitrogen N–(C)_3_, respectively.^11^ A small shift (of 0.1 eV) in the peak was observed in all Gd-doped samples after the addition of gadolinium atoms, which can be attributed to the bonding of Gd ions to N atoms to form Gd–N.^18^ From Fig. 4c, in the deconvoluted Gd 3d spectra, the two major peaks identified in the 1180–1230 eV region belong to Gd 3d_3/2_ and Gd 3d_5/2_ centered at 1220 and 1188 eV confirming the characteristic peak from core level electrons attributing to the Gd^3+^. The O 1s spectrum is observed in all the samples (Fig. S6†) between 530–535 eV which reveals the formation of –OH or C–O/C=O bonds. This is possibly due to the introduction of water during the synthesis process and also absorbed water and CO_2_ molecules from the atmosphere.^55–57^ The O 1s spectrum of exfoliated 10 wt% Gd-doped gCN sample (Fig. S7†) shows an additional peak at 529.4 eV corresponding to Gd–O bond.^58^ The formation of the Gd–O bond proves that the intercalating Gd atoms between the melon layers helped in the exfoliation and were exposed during the sonication process. Also, upon sonication, the Gd content of various samples is seen to decrease compared to its bulk counterpart (Table S2†) as some of the intercalating Gd atoms were lost during exfoliation while the rest of the Gd atoms remained attached to the surface of the exfoliated melon nanosheets. Exfoliation increases the sample's surface area, leading to higher oxygen content in the exfoliated melon nanosheets. The C/N ratio for gCN obtained from the wide survey spectrum was found to be 0.70, a value that is close to the corresponding ratio for melon-type carbon nitride (0.67).^59^ Interestingly after Gd doping, the ratio of C/N increases to 0.74, 0.76, 0.74, and 0.77 for GdgCN-1, GdgCN-3, GdgCN-5, and GdgCN-10, respectively. The increase in the C/N ratio occurs due to the preferential decomposition of nitrogen-rich compounds during the polymerization process in the form of ammonia, leaving behind carbon-rich residues.^60^ The sonication can also introduce defects leading to an increase in the overall C/N ratio. The comparison of deconvoluted C 1s, N 1s, O 1s, and Gd 3d in binding energies were summarized in Table S3.†

To confirm the doping of Gd atoms in melon-type carbon nitride using the solvent evaporation technique, we carried out an additional experiment by simply mixing bulk gCN with gadolinium nitrate. The mixture was sonicated for 2 h at 20% amplitude, and the material was washed with ethanol and nanopure water after centrifugation. The XPS measurements of the resulting sample before and after cleaning are displayed in Fig. S8.† The results show no significant Gd peaks in the washed sample, which suggests that the Gd atom was not doped in the gCN matrix *via* either interstitial or substitutional doping. Overall, the mixture of gadolinium nitrate to DCDA precursor using the solvent evaporation technique followed by thermal polymerization offers more stable doping.^61,62^

### 3.5. NMR studies

**Fig. 5:**
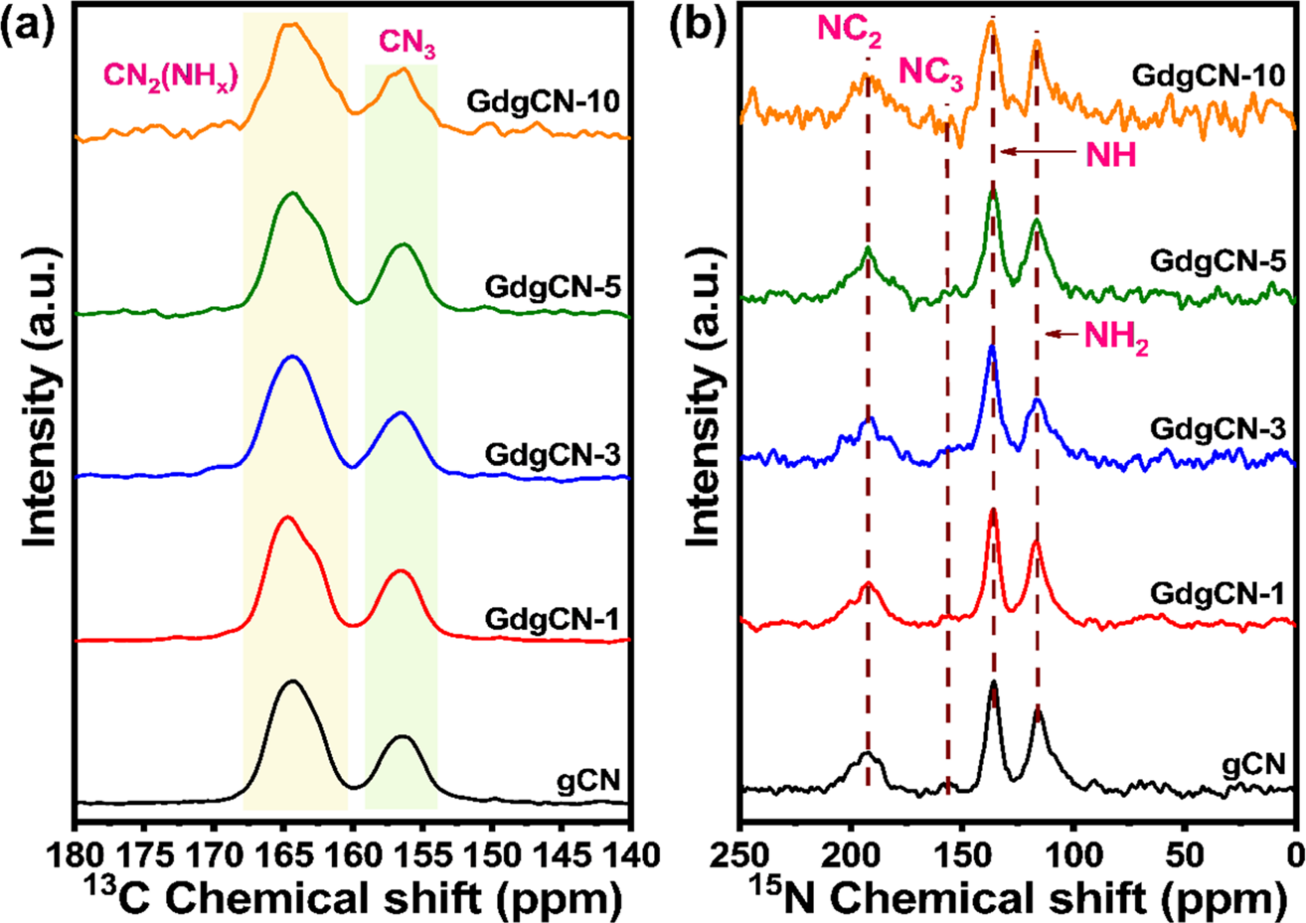
Solid-state (a) ^13^C and (b) ^15^N NMR spectra of gCN and Gd varied samples.

To examine the molecular structure at the atomic level of the prepared samples, solid-state NMR studies were carried out. As shown in Fig. 5a, ^13^C NMR spectra of the gCN sample display two sharp peaks at 164.38 and 156.29 ppm corresponding to the chemical shifts of NC_3_ and C_2_N-NH_*x*_ in the heptazine units, which is consistent with the previous reports.^63,64^ There are two overlapping peaks around 164 ppm (C_2_N (NH_*x*_)). The peak towards the left (∼165 ppm) is likely C_2_N (NH_2_), and the one on the right (∼163 ppm) is C_2_N (NH). The ^15^N NMR displays four peaks at 116.15, 136.08, 155.05, and 192.25 ppm (Fig. 5b).^65^ The signal at 192.2 ppm is attributed to NC_2_ (–N=C). The weak shoulder peaks at 155.05 ppm can be assigned to NC_3_. The two stronger peaks at 136.08 and 116.15 ppm were assigned to NH and NH_2_ groups, respectively. No new signals were observed upon the addition of Gd. This is expected due to fact that the relaxation of ^15^N nuclei will be so fast that it would be invisible in NMR. Upon increasing the Gd content the intensity of the signal gradually decreases revealing the gradual disintegration of the –CN_3_ structure, while the presence of NH and NH_2_ groups in ^15^N NMR suggests the formation of uncondensed or melon-type carbon nitride.^66,67^

NMR relaxation measurements were utilized to confirm the incorporation of the paramagnetic dopant into the lattice or structure. Direct proton relaxation measurement in the solid state is not feasible since considerable broadening of the proton resonances is seen even at a high spinning speed, and resolving individual components is impossible. Therefore, indirect observation of proton relaxation by ^13^C observation was used. In general, the proton spins within a single molecule are sufficiently strongly coupled by the homonuclear dipolar coupling that different relaxation is not seen for the different sites. As a result, one single average relaxation is observed for ^1^H. As shown in Table 1, a clear paramagnetic relaxation enhancement is seen as a function of Gd doping. This confirms the incorporation of Gd within the carbon nitride lattice.

**Table 1:** *T*_1_ values of gCN and GdgCN-*x* samples

Sample	^1^H *T*_1_ relaxation (s)
gCN	6.09
GdgCN-1	1.58
GdgCN-3	0.85
GdgCN-5	0.80
GdgCN-10	0.87

### 3.6. Computational analysis

The experimental results described in previous sections have indicated a number of essential differences in the binding modes of Gd to the melon framework. According to XPS data, in bulk melon, Gd is present as a Gd^(3+)^ species and bonding does not involve oxygen species or the formation of Gd–O bonds. Additionally, no GdO phase has been observed. Instead, formation of Gd–N bonds was suggested to take place based on the XPS results. Alternatively, when samples were prepared *via* sonication for the photoluminescence and UV-vis measurements, several changes were found. Morphological analysis indicated the presence of smaller and thinner size sheets upon increases in Gd concentration which was associated with an enhanced exfoliation of the samples. Different from the bulk case, the XPS analysis for these sonicated samples demonstrated the presence of additional peaks at 529.4 eV in the O 1s spectrum corresponding to the formation of Gd–O bonds. These findings indicate a change in the Gd bonding configuration for the exfoliated samples *vs.* the bulk doping case. Additionally, weakening of the Gd interaction with the melon framework was found to take place during sonication associated with a partial loss of the amount of doped Gd. Another piece of information was obtained based on NMR analysis which demonstrated the presence of Gd as a paramagnetic species and the possibility of having some disintegration of –CN_3_ structure with an increase in the amount of Gd doping. Finally, the results of photoluminescence and UV-vis measurements demonstrated the presence of a spectral blue shift upon Gd doping, corresponding to an increase in the *E*_opt_ values of the corresponding nanostructured materials. This latest finding is somewhat unexpected as doping of Gd as well as other rare earth trivalent ions was previously reported to lead to a reduction in the bandgap energy values. For example, La^3+^ decoration of two-dimensional g-C_3_N_4_ was found to decrease the bandgap from 2.83 to 2.21 eV with a corresponding increase in the photodegradation efficiency of ciprofloxacin under UV.^62^ Doping of Eu^3+^ in C_3_N_4_ was also found to lead to a narrowing of the bandgap from 2.62 eV for the undoped system to 2.53 eV for the doped one.^68^ Similarly, Li *et al.*^27^ in their study of Gd doping in g-C_3_N_4_ nanospheres with a bayberry-like structure have observed a decrease of the bandgap from 2.85 eV to 2.56 eV upon doping with 3% Gd leading to an increase in photocatalytic activity. A similar reduction in the HOMO–LUMO gap has been determined theoretically by Sarkar *et al.*^69^ for several trivalent rare-earth ions, *i.e.* Sm^3+^, Eu^3+^ and Gd^3+^ upon their adsorption on small graphitic carbon nitride clusters. A possible difference between these previous studies and the current work is the type of carbon nitride phase involved, *i.e.* the fully condensed C_3_N_4_ phase used in previous studies *versus* the melon phase considered in the current work but it is not clear if this phase variation is sufficient to explain the observed differences in energy gap changes upon Ln doping.

**Fig. 6:**
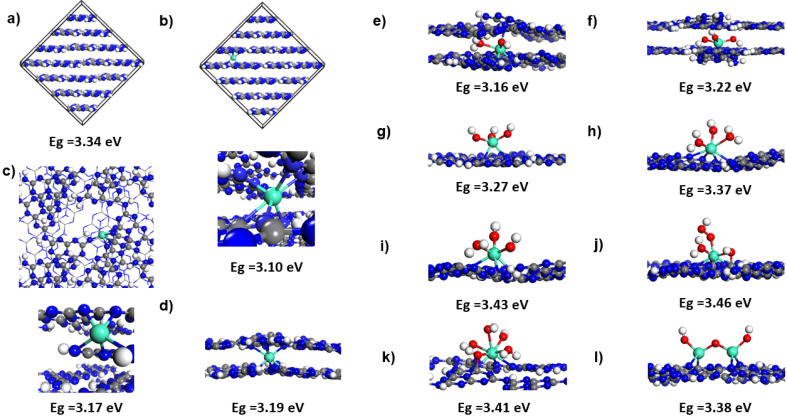
Pictorial view of melon bulk supercell (a) and various bulk (b and c), double layers (d–f) and single layer (g–l) melon structures where Gd doping has been considered. For O bonded species several combinations of OH, H_2_O or OOH species have been included as described in the text. In each case the corresponding electronic gap value is also indicated. The color scheme used is as follows: C (gray), N (blue), O (red), H (white) and Gd (light blue).

The different trends in the variation of the bandgap obtained in the previous studies^15,16,62,68^ and the one determined experimentally in the current work determined us to consider complementary theoretical investigations in an attempt to elucidate possible causes for the observed differences. For this purpose, we have performed a set of density functional theory calculations using 3D periodic models to elucidate the bonding of Gd both in bulk melon and on ultrathin melon sheets and to evaluate the corresponding changes in the electronic gap values. Guided by our experimental results where low Gd concentrations in bulk melon in the range of a few weight percent were involved, we have considered a large supercell model (see Fig. 6a) with a C_192_H_96_N_288_ composition in which a single Gd ion has been adsorbed (see Fig. 6b). The initial geometrical structure was taken as the heptazine-based graphitic g-C_6_N_9_H_3_ melon with *P*2_1_/*c* crystallographic symmetry as introduced by Mélissen *et al.*^29,39^ Consistent with our experimental results which showed a lack of Gd–O bonding or formation of a Gd–O phase upon doping, we assume that in bulk melon Gd adsorption takes place by direct bonding to framework atoms. For this purpose, in order to ensure the presence of Gd^(3+)^ species as indicated by XPS studies, a charge compensation has been achieved by considering the presence of H defects in the bulk. Preliminary test calculations have indicated that among different possible distributions of such H defects around the adsorbed Gd, the most stable configurations involve defects located at short separations from the Gd site and such an optimal distribution has been also considered for the model shown in panel (b) of Fig. 6. In a second model (see Fig. 6c), we analyze the possibility to have configurations that mimic the atomic structure after –CN_3_ dissolution as indicated by the results obtained based on NMR analysis. This has been done by using a bulk model (see Fig. 6c) in which two heptazine units in two nearby layers have been removed while Gd was doped around the resulting defect sites. Similar to the case described in Fig. 6b, three compensating H defects have been included to ensure charge neutrality for the entire system when Gd^(3+)^ species are present. For each of the three bulk structures represented in panels a–c of Fig. 6, *i.e.* bulk melon, bulk melon doped with Gd and bulk melon with two heptazine defective units doped with Gd, the corresponding bandgaps have been calculated and are indicated in the respective panels in Fig. 6. For bulk melon, a calculated gap of 3.34 eV was found which is in good agreement to results reported in previous work.^29,39^ From the structures depicted in panels (b) and (c) of Fig. 6, it can be seen that Gd intercalates in-between melon layers where it binds to adjacent layers *via* several Gd–N bonds mediated by the lone pair electrons of pyridinic nitrogen atoms. The calculated *E*_g_ for Gd-doped melon with or without the heptazine unit defect are both lower than the undoped, undefective melon structure. In particular, a 0.24 eV drop of the electronic gap from 3.34 eV to 3.10 eV is obtained upon Gd doping of melon. This variation range is similar to the one of 0.29 eV determined previously for Gd doping in g-C_3_N_4_ nanospheres.^27^ These findings demonstrate consistency between the calculated electronic gap variation obtained in this work for Gd doping in the melon phase and the previously reported results obtained for the fully condensed g-C_3_N_4_ phase.^27^

In order to address the observed blue spectral shift determined in our photoluminescence and UV-vis measurements we have considered a second set of configurations representing the case of ultrathin layers of melon. As indicated in previous sections, exfoliated layers can result from the preparation procedure during sonication, where direct interaction of Gd with oxygen species can take place. In order to simulate such a bonding environment, we have analyzed several different cases. These range from isolated Gd to Gd(OH)_*x*_ (*x* = 2,3) adsorbed in a bilayer system (see panels d–f in Fig. 6) or various Gd-bonded oxygen species, *i.e.* Gd(OH)_3_, Gd(OH)(H_2_O)_2_, Gd(OH)_2_(H_2_O), Gd(OH)(H_2_O)(OOH), Gd(OH)_3_(H_2_O)_2_ and Gd_2_O_3_ adsorbed on a single layer of melon (see panels e–l in Fig. 6). The diversity of these configurations is due to the possibility that upon adsorption of Gd(OH)_3_ or Gd_2_O_3_ species, additional H transfer from the edges of the heptazine melon units takes place leading to the formation of various combinations of H_2_O and OH species bonded to Gd. Alternatively, adsorbed O_2_ from the atmosphere on the Gd site in combination with H from the melon layer edges can lead to the formation of Gd-bonded OOH species. As seen from the data provided in Fig. 6, for all these Gd–O_*x*_ configurations adsorbed on bilayers or single layers of melon, the corresponding electronic gap values are all larger by as much as 0.36 eV relative to the 3.10 eV gap of Gd-doped bulk melon. Such variations of the electronic gap values can be responsible for the experimentally observed blue shift in optical measurements. The results obtained suggest that consistency between the trends obtained in the UV-vis measurements and the theoretical results is obtained if the samples obtained upon sonication in a liquid environment and then used in optical property measurements consist of exfoliated melon layers where Gd species can adsorb either as isolated species (within the melon bilayers) or as Gd–O_*x*_H_*y*_ species on ultra-thin bilayer or single layer systems. Formation of such species has been also indicated in XPS measurements where Gd–O bond formation was found for the sonicated samples. A final aspect analyzed is related to the paramagnetic nature of Gd^(+3)^ species when adsorbed in melon framework. This characteristic is confirmed by our calculations where a large magnetic moment of about 7 μ_B_ was found for each Gd atom. The distribution of magnetic density together with the corresponding density of states are shown in Fig. S9† for a subset of configurations. As can be seen in the represented cases, the spin density remains localized on Gd atoms independent of the specific bonding configuration. The presence of a large magnetic moment on Gd leads to an important split into majority and minority bands due to the exchange interaction as shown in the corresponding PDOS representations of Fig. S9.† Overall, the high magnetic moments of Gd doped in melon confirm the paramagnetic nature of Gd species as determined in our NMR measurements, making such systems attractive as high-performance catalysts or as contrast agents for magnetic resonance imaging applications.

## 4. Conclusion

In summary, we have synthesized a lanthanide-doped melon-type graphitic carbon nitride material from dicyandiamide and gadolinium precursors by a solvent evaporation method followed by direct thermal polymerization. We have found that this synthetic approach yields more stable doping of gadolinium ions within the resulting melon structure compared to post-synthetic metal ion intercalation. At the same time, our results reveal that the intercalated Gd species act as exfoliating agents assisting in forming thin melon nanosheets upon sonication. The effect of Gd doping on the structural, optical, and electronic properties of melon was studied experimentally using TEM, XRD, FTIR, UV-vis, PL, XPS, and NMR spectroscopy. XRD studies revealed mostly unperturbed melon crystal structure with small reduction of the long-range order along the *xy* plane due to the rupture of intralayer H-bonding upon gadolinium doping. XPS results confirmed the formation of Gd–N bonds in the bulk samples with Gd–O species observed in the sonicated samples. Additionally, the gadolinium-doped structures were investigated computationally by DFT studies. DFT calculations revealed atomistic level structure of Gd ion binding within bulk and few-layer melon-type carbon nitride structures as well as electronic bandgap values. The observed blue shift in UV-vis and PL spectra in the Gd-doped materials was rationalized by exfoliation of melon layers during the sonication process, which was confirmed by TEM as well as DFT calculations. The paramagnetic relaxation of the Gd-doped samples over pristine gCN at low doping percentages indicates the potential use of these materials for applications in magnetic resonance imaging (MRI). The combined experimental and theoretical study presented in this work on gadolinium doping of melon-type carbon nitride could pave the way for new applications of these hybrid materials.

## Data availability

All data are available in the paper and from the authors upon request.

## Author contributions

A. S. conceived the research project. G. K. performed materials synthesis and characterizations. D. C. S. performed DFT calculations of the gadolinium-doped carbon nitride structures. K. D. performed solid-state NMR experiments. R. A., S. E. C, and F. A. performed optical characterization of the materials. G. K. and D. C. S. drafted the manuscript. All authors read, edited, and approved the manuscript.

## Conflicts of interest

The authors declare that there is no conflict of interest.

## Supplementary Material

RA-014-D4RA03437F-s001
