# Supplementary material for: Influence of gadolinium doping on structural, optical, and electronic properties of polymeric graphitic carbon nitride
Source: RSC Adv. 2024 Jul 24;14(32):23342–51. doi: 10.1039/d4ra03437f (PMC11267507; doi:10.1039/d4ra03437f)
Supplement: RA-014-D4RA03437F-s001 [file RA-014-D4RA03437F-s001.pdf]

Electronic Supplementary Material (ESI) for RSC Advances.
This journal is © The Royal Society of Chemistry 2024

Supplementary Information

Influence of Gadolinium Doping on Structural, Optical, and Electronic Properties of Polymeric Graphitic Carbon Nitride

Ganesh Kesavan[a], Dan C. Sorescu[b,c], Raihan Ahamed[a], Krishnan Damodaran[a], Scott E. Crawford[b], Faezeh Askari[a], Alexander Star[a,d]*

[a] Department of Chemistry, University of Pittsburgh, Pittsburgh, Pennsylvania 15260, United States

[b] United States Department of Energy, National Energy Technology Laboratory, Pittsburgh, Pennsylvania 15236, United States

[c] Department of Chemical & Petroleum Engineering, University of Pittsburgh, Pittsburgh, Pennsylvania 15261, United States

[d] Department of Bioengineering, University of Pittsburgh, Pittsburgh, Pennsylvania 15261, United States

Corresponding author. Email: astar@pitt.edu

Table of Contents	
	Page Number
**Figure S1:** PL spectrum of gadolinium nitrate hexahydrate.	S-3
**Figure S2:** Plots of (a) emission maximum (nm), (b) FWHM (nm) against Gd doping.	S-3
**Figure S3:** Optical band gap energy (E_g) determination using the Tauc plot.	S-4
**Figure S4:** TEM images of bulk gCN and GdgCN-x samples.	S-5
**Figure S5:** Wide XPS spectrum of gCN and GdgCN-x samples showing C 1s, N 1s, O 1s, and Gd 3d elements.	S-5
**Figure S6:** High-resolution spectra of gCN and GdgCN-x samples showing O 1s.	S-6
**Figure S7:** High-resolution O 1s spectra of sonicated 10 wt% Gd-doped gCN.	S-7
**Figure S8:** High-resolution spectra of gCN and GdgCN samples showing Gd 3d.	S-7
**Figure S9:** Spin densities plots and projected density of states for selective Gd adsorption configurations	S-8
**Table S1:** Experimental λ_{max} values for gCN and GdgCN-x samples.	S-9
**Table S2:** XPS determined elemental composition of gCN and GdgCN-x samples.	S-9
**Table S3:** XPS determined C 1s, N 1s, O 1s, and Gd 3d in binding energies.	S-10

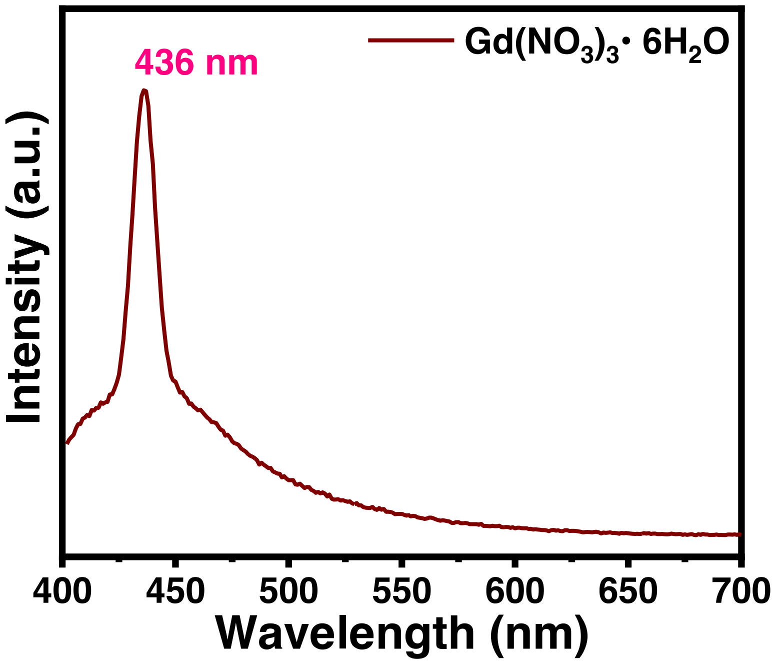

Figure S1: PL spectrum of gadolinium nitrate hexahydrate.

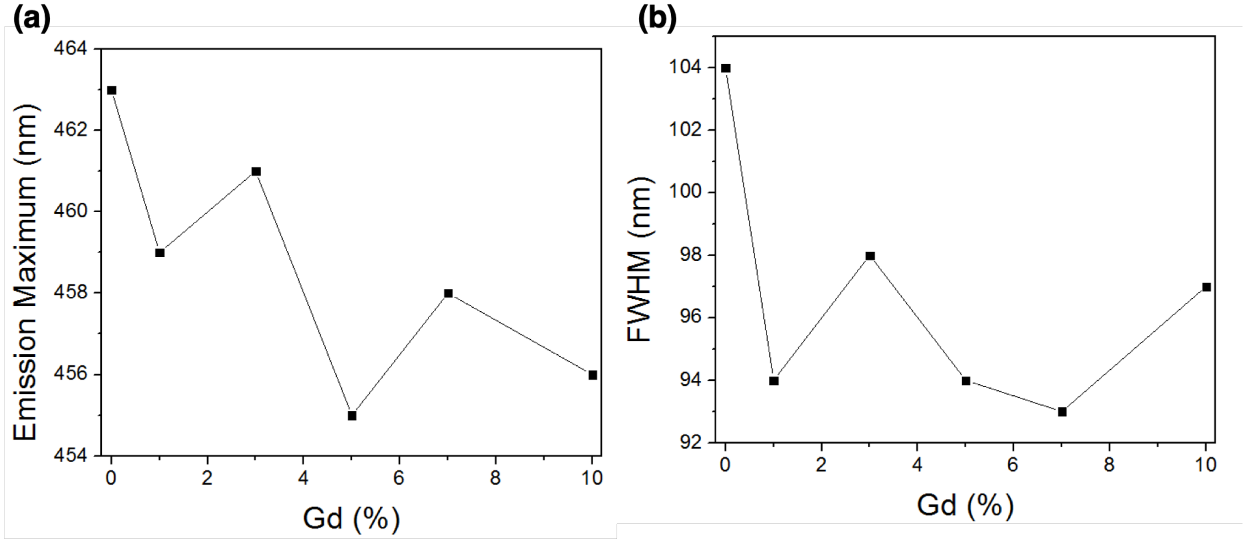

Figure S2: Plots of (a) emission maximum (nm), (b) FWHM (nm) against Gd doping.

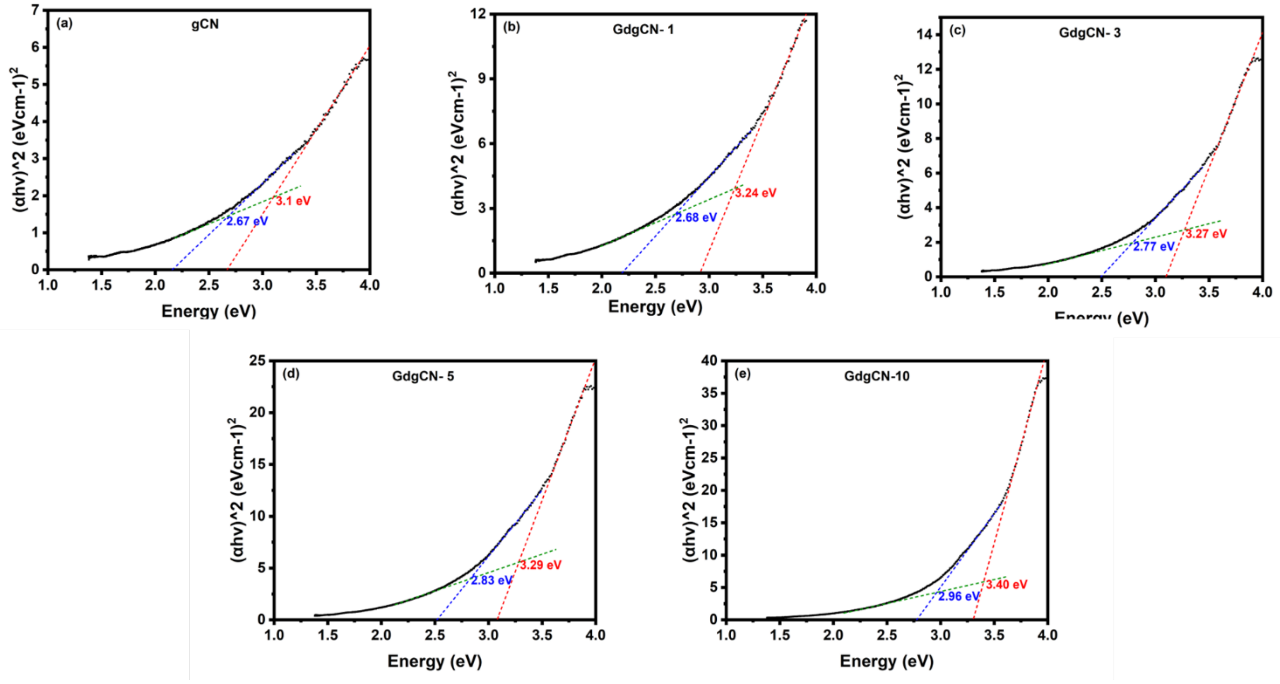

Figure S3: Optical bandgap energy (E_{opt}) determination of GdgCN-x samples using the Tauc plot. The numbers in red correspond to the bandgaps obtained from the fundamental absorptions. The numbers in blue correspond to the bandgaps obtained from the shoulder absorptions.

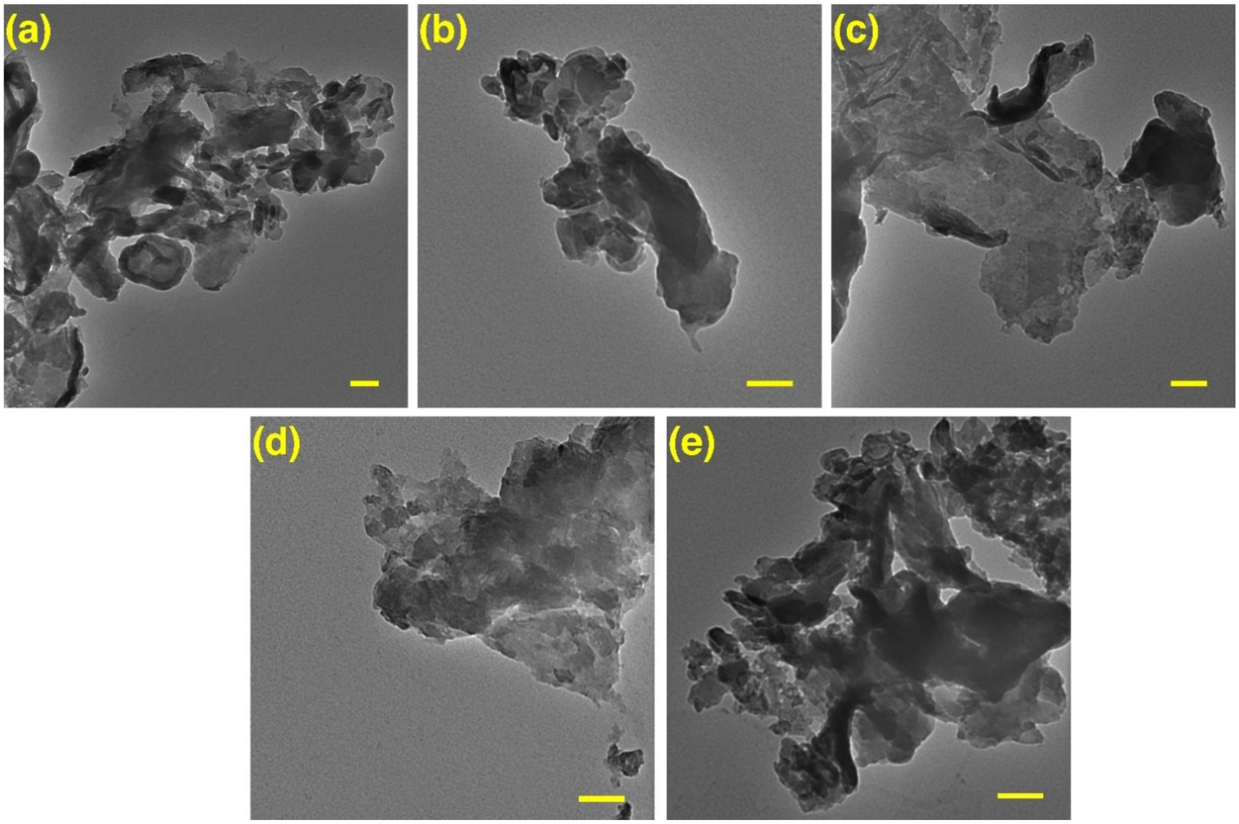

Figure S4: TEM images of bulk (a) gCN, (b) GdgCN-1, (c) GdgCN-3, (d) GdgCN-5, and (e) GdgCN-10 samples (Scale bar = 100 nm).

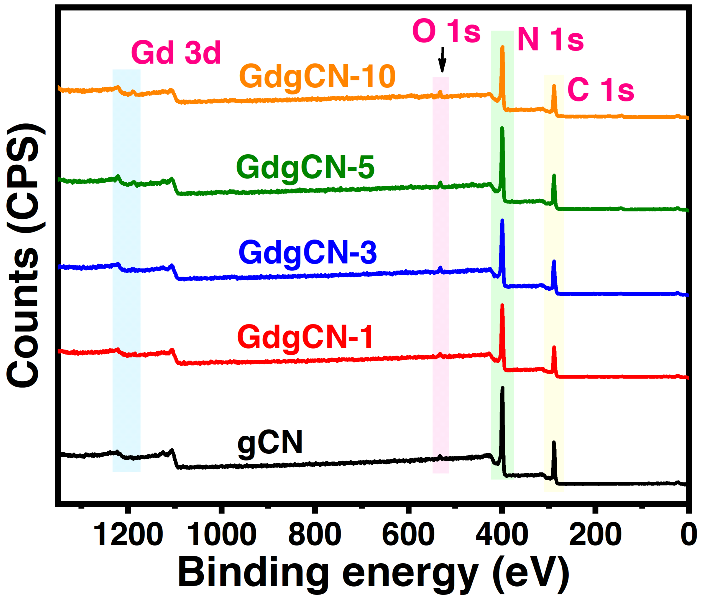

Figure S5: Wide XPS spectrum of gCN and GdgCN-x samples showing C 1s, N 1s, O 1s, and Gd 3d elements.

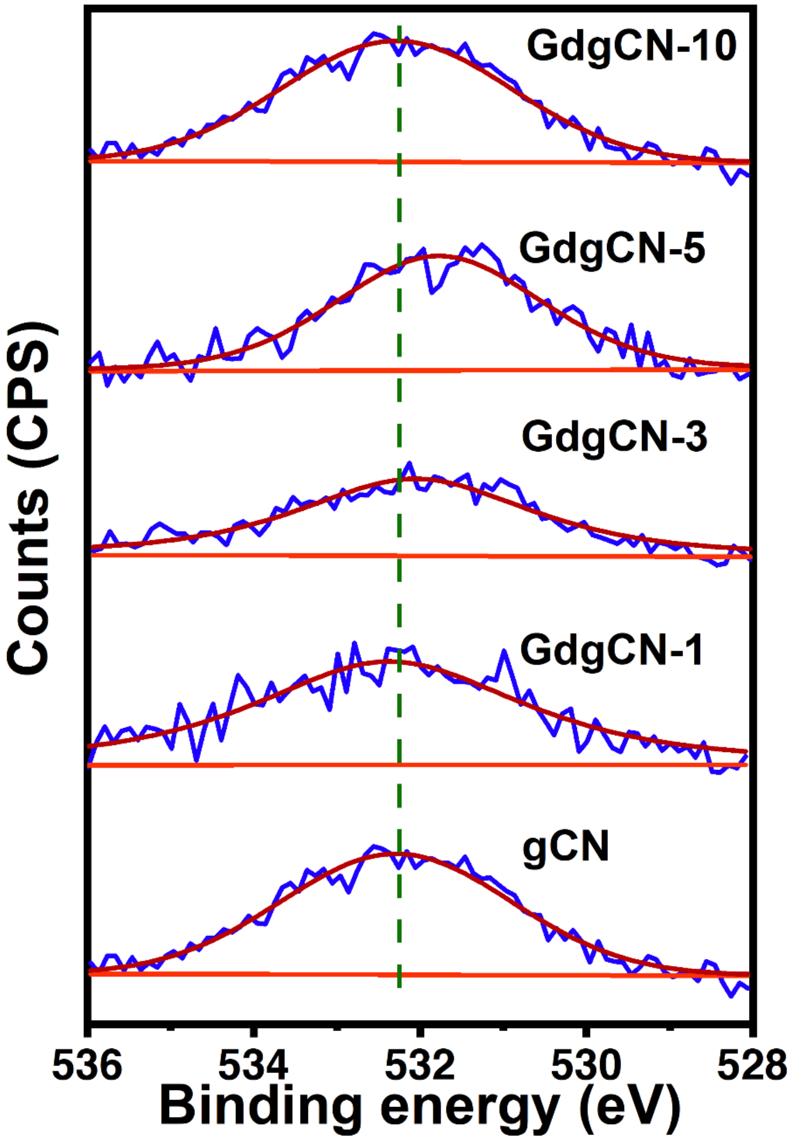

Figure S6: High-resolution spectra of gCN and GdgCN-x samples showing O 1s.

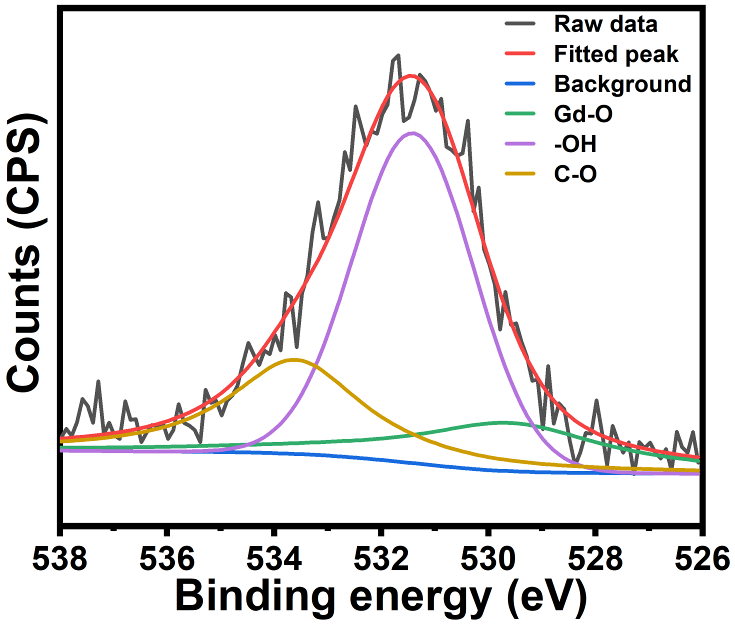

Figure S7: High-resolution O 1s spectra of sonicated 10 wt% Gd-doped gCN.

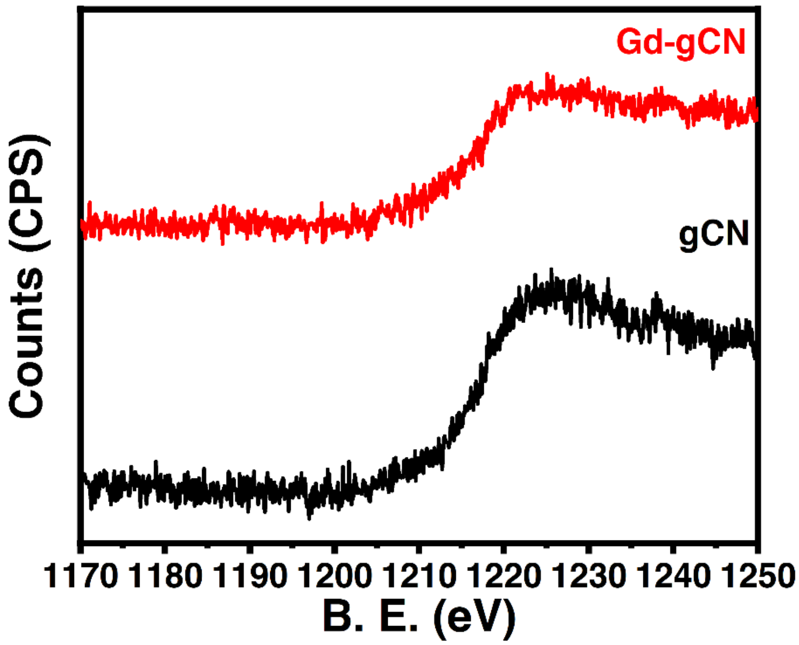

Figure S8: High-resolution spectra of gCN and GdgCN samples not showing any peaks in the Gd 3d region.

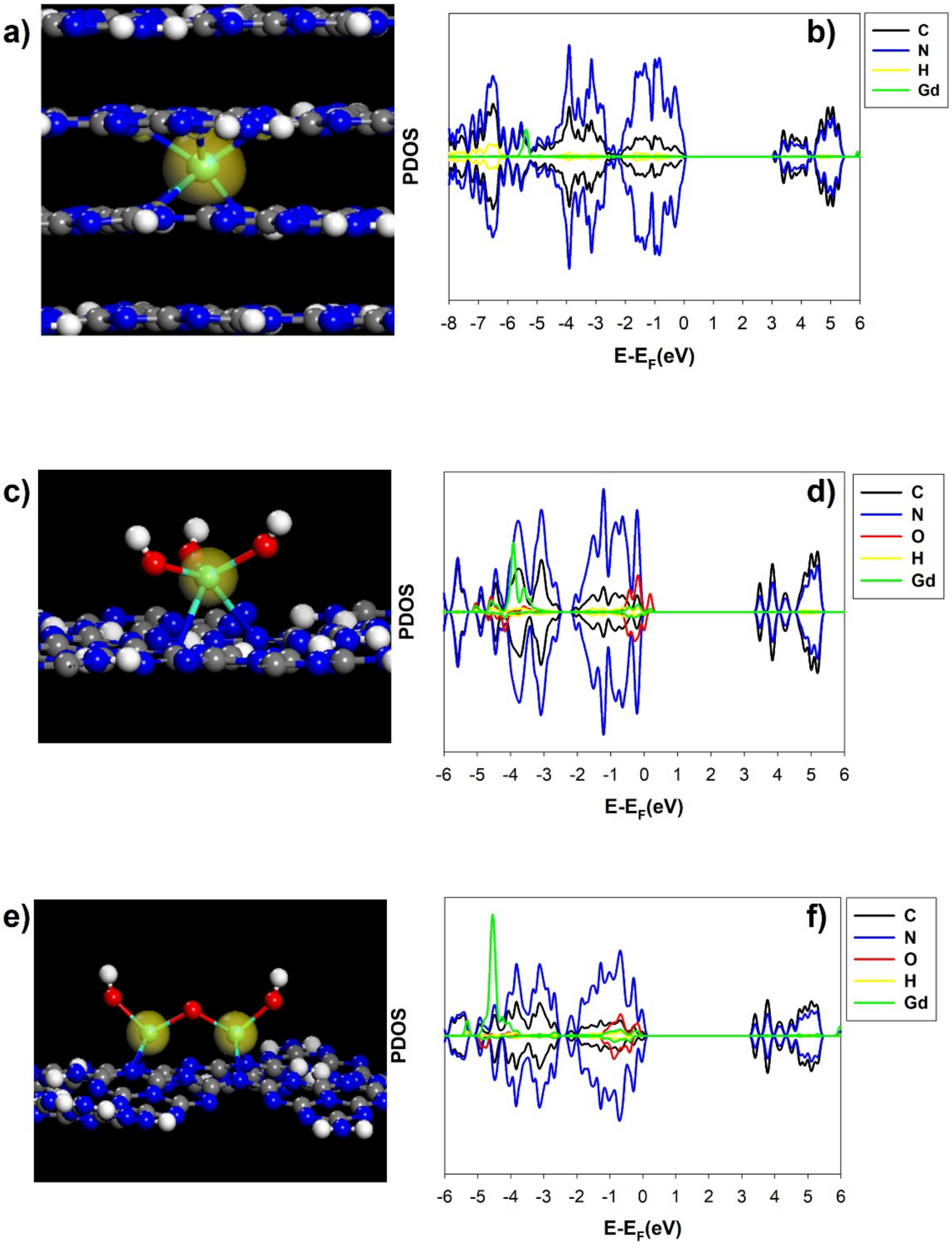

Figure S9. Spin densities plots and projected density of states for Gd adsorbed in bulk melon (a), (b), and for Gd-$(OH)_3$ (c), (d) and Gd_2O_3 (e), (f) species adsorbed on a single layer melon structure. In panel (e), two of the H atoms are transferred from the melon edge to the adsorbed Gd_2O_3 cluster.

**Table S1:** Experimental λ_{max} values for gCN and GdgCN-x samples.	
**Sample**	**λ_{max} (nm)**
gCN	463
GdgCN-1	459
GdgCN-3	461
GdgCN-5	455
GdgCN-10	456

**Table S2:** XPS determined elemental composition of gCN and GdgCN-x samples.					
**Samples**	**C 1s (at %)**	**N 1s (at %)**	**O 1s (at %)**	**$Gd_{3/2}$ (at %)**	**$Gd_{5/2}$ (at %)**
gCN	40.71	57.66	1.64	0	0
GdgCN-1	39.82	57.38	2.71	0.06	0.03
GdgCN-3	42.99	53.4	3.47	0.05	0.09
GdgCN-5	40.29	56.36	2.8	0.35	0.2
GdgCN-10	41.84	54.03	3.4	0.45	0.29
Exfoliated GdgCN-10	43.91	47.06	8.74	0.24	0.18

Table S3: XPS determined C 1s, N 1s, O 1s, and Gd 3d in binding energies.				
**Sample**	**C 1s (eV)**	**N 1s (eV)**	**O 1s (eV)**	**Gd 3d (eV)**
gCN	284.84<br>288.13<br>293.74	398.63<br>399.58<br>400.48<br>401.32<br>404.75	532.44	0
GdgCN-1	284.74<br>288.04<br>293.64	398.42<br>399.55<br>400.39<br>401.03<br>404.22	532.36	1187.28<br>1220.92
GdgCN-3	284.80<br>288.25<br>293.77	398.43<br>399.52<br>400.27<br>401.1<br>404.39	532.09	1187.60<br>1222.15
GdgCN-5	284.8<br>288.21<br>294.05	398.45<br>399.39<br>400.33<br>401.12<br>404.80	531.77	1188.15<br>1222.57
GdgCN-10	284.81<br>288.02<br>293.85	398.38<br>399.55<br>400.34<br>401.14<br>405.65	532.28	1188.52<br>1223.05
